# A cmap-enabled gene expression signature-matching approach identifies small-molecule inducers of accelerated cell senescence

**DOI:** 10.1186/s12864-019-5653-x

**Published:** 2019-04-15

**Authors:** Ding Wu, Brett Pepowski, Satoe Takahashi, Stephen J. Kron

**Affiliations:** 1 Department of Molecular Genetics and Cell Biology and Ludwig Center for Metastasis Research, The University of Chicago, 929 East 57th Street, GCIS W522A, Chicago, IL 60637 USA; 2 Department of Otolaryngology - Head and Neck Surgery, Feinberg School of Medicine, Northwestern University, Chicago, IL 60611 USA

**Keywords:** Radiation-induced senescence, p21-induced senescence, Transcriptome analysis, Differential expression genes, DNA damage foci, Upstream regulator factors, Connectivity map analysis, Small molecule inhibitors

## Abstract

**Background:**

Diverse stresses including genotoxic therapy can induce proliferating cancer cells to undergo cellular senescence and take on the characteristic phenotypes of replicative cellular aging. This accelerated or therapy-induced senescence has been alternatively proposed to contribute to therapeutic efficacy or resistance. Toward better understanding this cell state, we sought to define the core transcriptome of accelerated senescence in cancer cells.

**Results:**

We examined senescence induced by ionizing irradiation or ectopic overexpression of the stoichiometric cyclin-dependent kinase (CDK) inhibitor p21^CIP/WAF1/SDI1^ in the human breast cancer cell line MCF7. While radiation produces a strong DNA damage response, ectopic expression of p21 arrests cell cycle progression independently of DNA damage. Both conditions promoted senescence within 5 days. Microarray analysis revealed 378 up- and 391 down-regulated genes that were shared between the two conditions, representing a candidate signature. Systems analysis of the shared differentially expressed genes (DEGs) revealed strong signals for cell cycle control and DNA damage response pathways and predicted multiple upstream regulators previously linked to senescence. Querying the shared DEGs against the Connectivity Map (cmap) database of transcriptional responses to small molecules yielded 20 compounds that induce a similar gene expression pattern in MCF7 cells. Of 16 agents evaluated, six induced senescence on their own. Of these, the selective estrogen receptor degrader fulvestrant and the histone acetyltransferase inhibitor vorinostat did so without causing chromosomal damage.

**Conclusions:**

Using a systems biology approach with experimental validation, we have defined a core gene expression signature for therapy-induced senescence.

**Electronic supplementary material:**

The online version of this article (10.1186/s12864-019-5653-x) contains supplementary material, which is available to authorized users.

## Background

From its initial observation by Hayflick and Moorhead [1], the limited proliferative capacity of normal cells in culture and their terminal arrest via replicative senescence has been considered the cellular equivalent of organismal aging. Replicative senescence (RS) has long been attributed to telomere erosion but may also reflect mitochondrial and chromosomal replication stress and other effects of accumulated oxidative damage [2]. Stress-induced premature senescence (SIPS) can be triggered by genotoxic stress, oxidative stress, oncogenic stress, chromatin disruption and other acute stresses [3–6]. Therapy-induced senescence (TIS) is observed in normal and tumor cells following radiation and/or chemotherapy. Programmed senescence is a recently described feature of early development [7]. Common features of these diverse forms of senescence include enlarged cell size, unrepaired genomic damage, proliferative arrest linked to expression of the p21^CIP/WAF1^ and/or p16^Ink4A^ stoichiometric cyclin-dependent kinase inhibitors, and activation of senescence-associated β-galactosidase activity (SA-β-Gal) [8–12].

Current understanding is that a common initiating event for senescence is accumulation of unrepairable DNA damage such as eroded telomeres, collapsed replication forks or chromosomal double strand breaks (DSBs). This places senescence as an endpoint of the DNA damage checkpoint pathway, where p53-dependent p21 expression initiates cell cycle arrest. Nonetheless, what directs cells toward senescence rather than cell death, autophagy or a return to proliferation remains poorly understood. Once formed, senescent cells can persist indefinitely and actively perform autocrine and paracrine signaling, which has been implicated in inflammatory responses, fibrosis, carcinogenesis and other changes in proliferation and gene expression. This senescence-associated secretory phenotype (SASP) appears to depend on DNA damage-induced, NF-κB-dependent expression of inflammatory cytokines, chemokines, and growth factors [13]. Separating the senescence and SASP pathways, ectopic expression of p21 or p16 can induce senescence without DNA damage or a SASP [14] and targeting NF-κB, PARP, HMG-CoA reductase, p38, JAK, MLL1, BRD4 or other targets can suppress the SASP without blocking senescence after DNA damage [15–18]. Senescence itself can be blocked by protecting telomeres from erosion, limiting other DNA damage, mitigating oxidative stress or disrupting downstream signaling [19, 20]. There is a complementary interest in restoring replicative senescence in cancer by blocking telomere maintenance or promoting therapy-induced senescence after genotoxic cancer therapy [21, 22]. This has stimulated a recent focus by our group and others toward identifying agents that promote cancer cell senescence on their own or in combination with other genotoxic stresses [23, 24].

A weakness shared by studies to date is that while pathways upstream of the SASP are now well-defined, the mechanism of senescence itself remains obscure, meaning that specific targets have yet to be identified. We reasoned that along with a characteristic cellular phenotype, senescent cells may display a distinctive gene expression signature independent of whether the SASP is expressed or not. Thus, we have compared the transcriptomes of senescent MCF7 human mammary carcinoma cells formed by radiation or overexpression of the p21 protein to generate a candidate senescence signature. Systems analysis flagged mostly pathways associated with cell cycle arrest, offering minimal insight, although several upstream signaling regulators were identified that may determine specific features of senescent cell arrest. Toward identifying new chemical probes for senescence, we queried the Connectivity Map (cmap) database of microarray expression data from cell lines treated with bioactive small molecules [25]. Of the 20 highest scoring gene expression patterns, we examined 16 compounds in vitro and identified four that promote senescence along with DNA damage foci and two others that appear to phenocopy p21 overexpression by inducing senescence without DNA damage foci. This work provides a pathway toward defining a core gene expression signature for senescence and leveraging this pattern to discover candidate chemical probes to dissect critical pathways.

## Methods

### Cell lines and cell culture

Both cell lines MCF7^GFP-IBD^ and MCF7^p21^ were generated in our laboratory through modification of the MCF7 Tet-On Advanced cell line (Clontech). The generation and characterization of the MCF7^GFP-IBD^ cell line has been described [26]. Briefly, GFP fused to the human 53BP1 ionizing-radiation induced foci binding domain (IBD) was cloned and transduced into the MCF7 Tet-On Advanced cell line (Clontech). MCF7^p21^ was developed from MCF7-FUCCI cells into which an inducible p21 construct was transduced. Briefly, MCF7-FUCCI cells were generated by lentiviral transduction of MCF7 Tet-On cells with FUCCI constructs [27] followed by cell sorting. To express p21 in MCF7-FUCCI, the wild-type (WT) CDKN1A open reading frame (p21, Origene NM_078467) [28] from human cDNA was PCR amplified with primers *5′*CGACGGATCCATGTCAGAACCGGCTGGGGATGTCCGTCAG*3′* and *5′*CGACGAATTCTTAGGGCTTCCTCTTGGAGAAGATCAGCCG*3′*. PCR products were then digested with BamHI and EcoRI, and cloned into pLVX-Tight-Puro (Clontech) to obtain pLVX-p21. pLVX-p21 lentivirus was prepared using packaging mix (Clontech) and transduced into MCF7-FUCCI Tet-On Advanced, followed by puromycin selection at 0.5 μg/ml to obtain MCF7^p21^ cells.

Cell lines were maintained in Dulbecco’s Modified Eagle Medium (DMEM) with 4 mM L-glutamine (Invitrogen), supplemented with 10% Tet system approved FBS (Clontech) and penicillin-streptomycin (Pen-Strep, Thermo) at 5% carbon dioxide and 37 °C. MCF7^p21^ and MCF7-FUCCI cells were maintained with 0.5 μg/ml puromycin.

### Immunofluorescence and IR-induced foci (IRIF) imaging

For immunofluorescence, cells were fixed with 2% paraformaldehyde (PFA) and stained with anti-phospho-γH2AX (Millipore, clone JBW301) detected with anti-mouse Alexa 488 (Jackson) or anti-p21 (Thermo) detected with anti-rabbit Alexa 647 (Jackson). Images were captured on a Zeiss Axiovert 40 CFL microscope with a 40X Plan-Neofluar objective and Axiocam digital camera controlled by AxioVision 4.8 software. Images were pseudo-colored in Adobe Photoshop or ImageJ (http://imagej.nih.gov/ij/). For IRIF imaging, MCF7^GFP-IBD^ was induced with 1 μg/ml doxycycline (Sigma) for 24 h. On the second day cells were irradiated with 6 Gy of ionizing radiation (IR) or treated with compounds. Cells were fixed with 2% PFA and foci were imaged as previously described [19] .

### SA-β-Gal senescence assay

The SA-β-Gal assay was performed as described previously [12] with minor modification [19]. Briefly, cells were seeded on microscope cover slips in 24-well plates at 1 × 10^4^ per well. After 1 day, cells were treated with 10 μM compounds and cultured for 5 more days. On the sixth day, cells were fixed with 2% PFA for 5 min and washed with PBS. Then the cells were incubated at 37 °C (without CO_2_) in staining solution consisting of 1 mg/ml X-gal (5-bromo-4-chloro-3-indolyl β-D-galactopyranoside) in 40 mM citric acid, pH 6.0, 3.3 mM potassium ferrocyanide, 3.3 mM potassium ferricyanide, 150 mM NaCl and 20 mM MgCl_2_. Staining lasted for 4–8 h, until color became evident. Images were captured on a Zeiss Axiovert 200 M microscope with 20X Plan-NeoFluar objective and Axiocam digital color camera controlled by AxioVision Rel 4.8 software.

### RNA extraction, Illumina array hybridization and transcriptome analysis

RNA was extracted using Trizol (Invitrogen) and further purified using RNeasy mini kits (Qiagen). 1 μg total RNA was used for generation of cRNA which was hybridized to Illumina Microarray HumanHT12. All cRNA probes, oligonucleotide microarray manipulations, and scanning of arrays were carried out by the University of Chicago Functional Genomics core facility (https://fgf.uchicago.edu/). Data were analyzed by Illumina GenomeStudio (background correction, quantile normalization) and filtered by *p* ≤ 0.05. Differentially expressed genes were detected with Significance Analysis of Microarrays (SAM) [29] with a 1.5 fold cutoff and false discovery rate of 0.01. A Python program was written to collapse genes based on average of probes (Additional file 1).

### Gene Expression Pathway analysis

Ingenuity Pathway Analysis (IPA, https://www.qiagenbioinformatics.com/products/ingenuity-pathway-analysis/) was applied for functional pathway analysis. Canonical Pathway Analysis identified the pathways from the Ingenuity Pathways Analysis library of canonical pathways that were significantly enriched. The significance of the association between the uploaded dataset and the canonical pathway was measured in ratio and *p*-value from Fisher’s exact test. In Database for Annotation, Visualization and Integrated Discovery (DAVID) (https://david-d.ncifcrf.gov/) [30] Kyoto Enrichment of Genes and Genomics (KEGG) [31] pathways analysis was applied. Enriched Gene Ontology (GO) terms in cellular process were analyzed through GOrilla (http://cbl-gorilla.cs.technion.ac.il/) [32] and REVIGO (http://revigo.irb.hr/) [33].

### Connectivity Map analysis

Connectivity Map build02 [25] was used, containing more than 7000 expression profiles, representing 1309 small molecules, available at http://www.broad.mit.edu/cmap/. Since cmap uses the Affymetrix platform, we first changed the gene list from gene symbol to Affymetrix probe through Affymetrix batch query. Then the obtained probe list as a query signature (include two lists consisting of ~ 500 up-regulated gene probes and ~ 400 down-regulated gene probes) was used to run against the cmap database to generate hits. In cmap, a “perturbagen” is defined as any small molecule or genetic reagent. The similarity between the gene expression profile of the query signature and that of a cmap instance is measured by the connectivity score, ranging from − 1 to 1. A nonparametric, rank-based pattern matching strategy based on Kolmogorov-Smirnov statistics was used to determine connectivity score and *p*-value.

## Results

### Radiation-induced and p21-mediated senescence

**Fig. 1:**
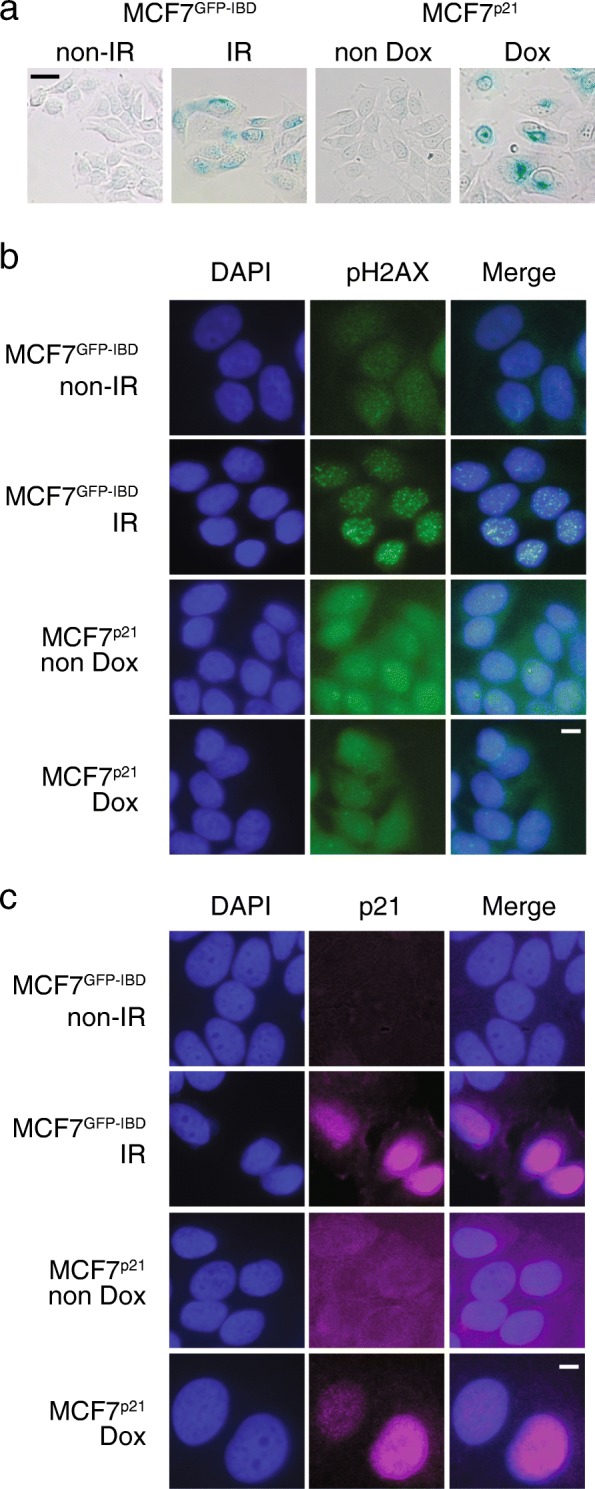
Radiation and p21 overexpression induce similar senescence phenotypes. **a** SA-β-gal assay of MCF7^GFP-IBD^ cells, 5 days after 6 Gy irradiation, compared to MCF7^p21^ cells, 5 days after induction with doxycycline, displays activation of beta-galactosidase activity (blue staining) under both conditions. Scale bar = 20 μm. **b** Immunofluorescence staining for phospho-H2AX in MCF7^GFP-IBD^ cells demonstrates nuclear foci 3 h after 6 Gy irradiation while MCF7^p21^ cells display no foci 3 h after induction with doxycycline. Scale bar = 20 μm. **c** Immunofluorescence staining for p21 in MCF7^GFP-IBD^ cells, 5 days after 6 Gy irradiation, compared to MCF7^p21^ cells, 5 days after induction with doxycycline, demonstrates increased expression under each condition. Untreated controls are shown for each assay. Scale bar = 20 μm.

In this study, we reexamined accelerated senescence triggered by ionizing radiation (IR) compared to ectopic expression of p21. We used MCF7^GFP-IBD^ cells [26] expressing GFP fused to the 53BP1 binding domain (GFP-IBD), to track ionizing radiation induced foci (IRIF) formation at DNA double strand breaks (DSBs). Along with the GFP-IBD foci, we also used immunofluorescence detection of phospho-H2AX (γH2AX) as a second DSB reporter. To study senescence triggered by ectopic expression of p21, we constructed MCF7^p21^ by transduction of MCF7 cells with a lentivirus encoding a Tet-On regulated CDKN1A (p21) gene. To induce senescence, MCF7^GFP-IBD^ cells were irradiated with 6 Gy and MCF7^p21^ cells were treated with 1 μg/ml doxycycline. After 5 days, both cell lines adopted characteristic features of senescent cells including enlarged cell and nuclear size, a flattened morphology, and increased SA-β-Gal staining compared to untreated controls (Fig. 1a). As expected, only the irradiated cells displayed increased γH2AX foci while cells overexpressing p21 showed no similar effects (Fig. 1b). Immunofluorescence with anti-p21 revealed increased nuclear expression in both the irradiated MCF7^GFP-IBD^ and doxycycline-induced MCF7^p21^ cells at 5 days (Fig. 1c).

### Functional gene expression studie

**Fig. 2:**
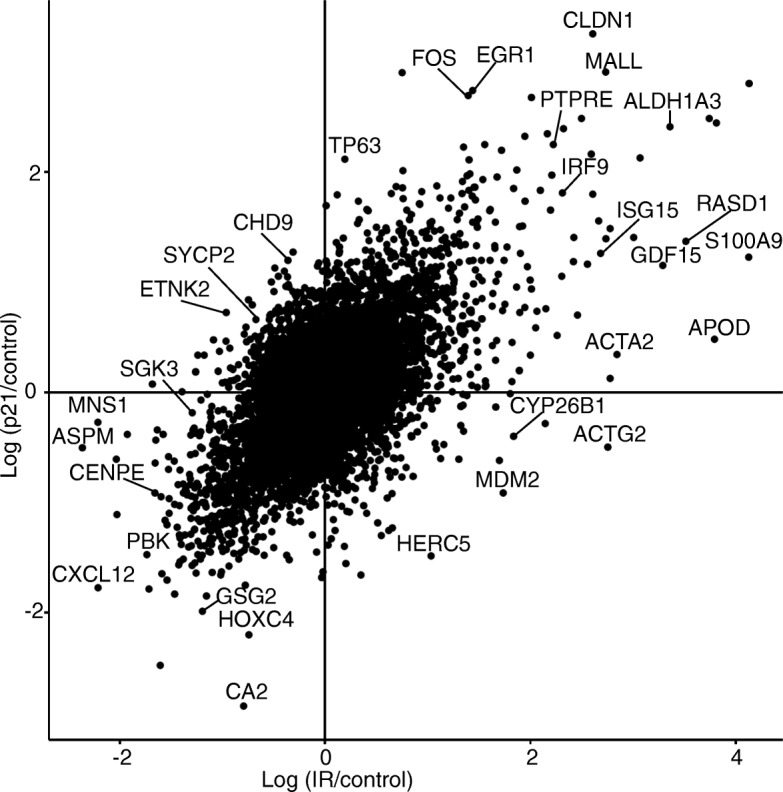
Radiation- and p21-induced senescence display related gene expression patterns. Plot shows differentially expressed genes detected in radiation-induced and/or p21-induced senescence. Microarray analysis was performed comparing of MCF7^GFP-IBD^ cells, 5 days after 6 Gy irradiation, or MCF7^p21^ cells, 5 days after induction with doxycycline, to untreated controls. The relative expression of all detected genes is shown, log transformed. Selected genes are labeled.

**Fig. 3:**
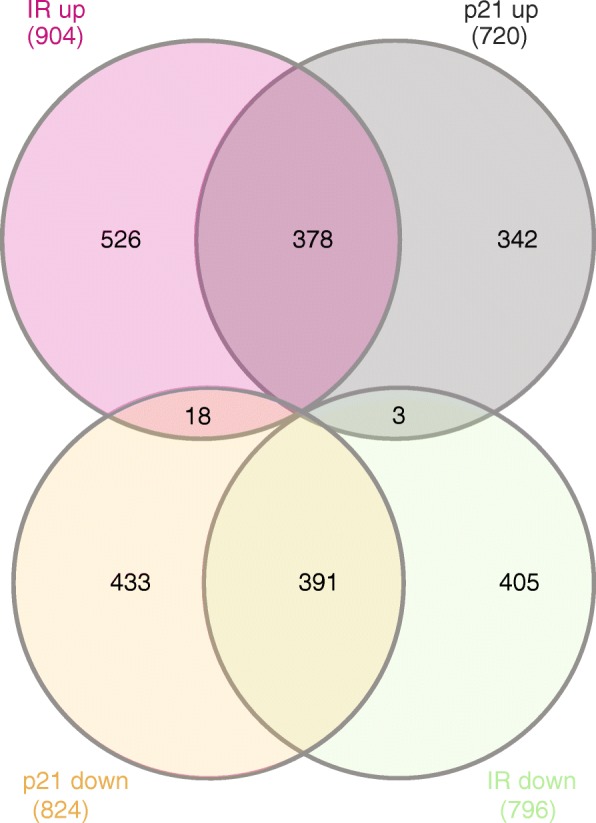
A potential senescence signature defined by altered gene expression after radiation and p21 overexpression. Venn Diagram analysis of the differentially expressed genes (DEGs) identified by microarray analysis of MCF7^GFP-IBD^ cells, 5 days after 6 Gy irradiation, compared to MCF7^p21^ cells, 5 days after induction with doxycycline, determined by comparison to untreated controls. Shown are numbers of up- or down-regulated genes with fold change >1.5. Note high overlap between DEGs, with ~50% of all genes regulated by p21 also regulated by radiation, and very few displaying reciprocal expression.

Toward identifying a shared pattern of differentially expressed genes (DEGs) between radiation-induced and p21-mediated senescence, we performed microarray transcriptome analysis. RNA was obtained from irradiated MCF7^GFP-IBD^ and doxycycline-induced MCF7^p21^ cells after 5 days incubation along with untreated controls. Samples were reverse transcribed, fluorescently labeled, combined in pairs to their respective controls, hybridized to Illumina Human HT12 chips and analyzed with GenomeStudio. The radiation-induced senescent cells yielded 15,964 transcript probes at a detection *p*-value less than 0.05 while the p21-induced sample yielded 15,936 probes. SAM analysis to identify differentially expressed genes at FDR = 0.01 and fold change ≥1.5 identified 1976 transcript probes in radiation senescent cells and 1819 in p21 senescent cells. Averaging fold expression between multiple probes identifying a common gene resulted in 1701 DEGs for the radiation sample and 1544 for the p21 sample, of which 790 were shared. Plotting the fold expression in radiation- versus p21-induced senescence of all genes showed many genes that displayed similar differential regulation (Fig. 2). Among common DEGs, 769 of 790 (97%) showed the same direction of change, with 391 downregulated and 378 upregulated (Fig. 3, Additional file 2), consistent with the expression pattern of components of a senescence signature. Toward systems level analysis, the radiation-induced DEGs, p21-induced DEGs and the 769 shared DEGs were subjected to Ingenuity Pathway Analysis (IPA, https://www.qiagenbioinformatics.com/products/ingenuity-pathway-analysis/), KEGG pathways study in Database for Annotation, Visualization and Integrated Discovery (DAVID) [31] and visualization in REVIGO [33].

IPA analysis of the shared DEGs yielded 12 canonical pathways at a cut-off log (*p*-value) of 2 (*p*-value ≤0.03, Table 1), with several related to DNA damage response and cell cycle, including *Role of BRCA1 in DNA Damage Response*, *Role of CHK Proteins in Cell Cycle Checkpoint Control*, *Mitotic Roles of Polo-Like Kinase*, and *Cell Cycle: G2/M DNA Damage Checkpoint Regulation*. Among genes that flagged these pathways were multiple downregulated cell cycle genes including transcription factor E2F, cyclins CCNB1 and CCNB2, cyclin-dependent kinase CDK1, replication factor C components RFC2, RFC3, RFC4, and RFC5, MCM replication factors MCM5 and MCM7, and other regulators of mitosis and cell division including PLK1, CDC25C, and CDC20. IPA analysis also identified three pathways that distinguished radiation-induced senescence from p21-induced senescence, *Interferon Signaling*, H*epatic Fibrosis/Hepatic Stellate Cell Activation*, and *Death Receptor Signaling Leading to Apoptosis*. In the interferon pathway, genes IFNGR1, STAT1, IRF9, IRF1, IFITM1, IFITM3 were specifically upregulated in the radiation-induced senescent cells (Additional file 3).

**Table 1:** Most significant canonical pathways identified by IPA analysis of the shared differentially expressed genes (DEGs)

Ingenuity canonical pathways	-log(*p*-value)
Role of BRCA1 in DNA Damage Response	8.54
Role of CHK Proteins in Cell Cycle Checkpoint Control	8.05
Mitotic Roles of Polo-Like Kinase	7.54
Cell Cycle: G2/M DNA Damage Checkpoint Regulation	6.06
Cell Cycle Control of Chromosomal Replication	5.41
ATM Signaling	5.22
Mismatch Repair in Eukaryotes	5.2
Hereditary Breast Cancer Signaling	4.63
Estrogen-mediated S-phase Entry	4.58
BER pathway	3.56
Cyclins and Cell Cycle Regulation	2.91
β-alanine Degradation I	2.52

Much like the results of IPA analysis, applying DAVID to the 769 shared DEGs yielded 10 KEGG (Kyoto Enrichment of Genes and Genomics) pathways that were linked to DNA replication, cell cycle progression and DNA repair (Table 2).

**Table 2:** Most significant KEGG pathways enriched in the shared differentially expressed genes

Term	Gene count	*P*-value
DNA replication	17	2.00E-13
Cell Cycle	28	4.10E-13
Oocyte meiosis	18	2.10E-06
Base excision repair	10	7.60E-06
Mismatch repair	8	3.00E-05
Nucleotide excision repair	9	4.20E-04
p53 signaling pathway	10	2.00E-03
Progesterone-mediated oocyte maturation	11	2.90E-03
Homologous recombination	5	2.90E-02
Pyrimidine metabolism	9	4.40E-02

**Fig. 4:**
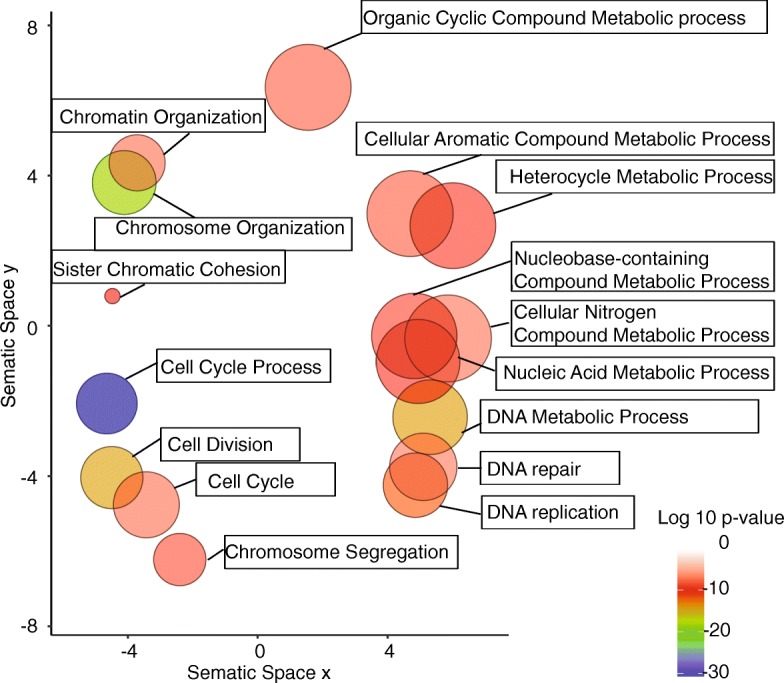
Gene ontology analysis identifies cell proliferation pathways in the senescence signature. The DEGs shared between radiation- and p21-induced senescent cells were subjected to GO enrichment analysis, then summarized and visualized as a scatter plot using the REVIGO Web server. GO terms related to cellular process are represented by circles and are plotted according to semantic similarities to other GO terms (adjoining circles are most closely related). Circle size is proportional to the frequency of the GO term, while color indicates the log10 *p* value (*red* higher, *blue* lower). GO terms with a log10 *p* value of −7 or less for cellular process are labeled.

We also examined enrichment of Gene Ontology (GO) classes with GOrilla and REVIGO. The 769 shared DEGs were ranked from most down-regulated to most up-regulated based on their expression in the radiation-treated cells and input to GOrilla, yielding 18 cellular process GO terms with cut-off *p*-value ≤10^− 7^ mostly linked to DNA repair, replication and recombination, chromosome segregation and nucleic acid metabolism (Fig. 4).

### Analysis of upstream regulators by IPA

While systems level analysis of the 769 common DEGs identified multiple pathways linked to the proliferative arrest characteristic of senescence, it failed to reveal unanticipated determinants of the senescent cell phenotype. As an alternative strategy, we re-examined the radiation- and p21-associated DEGs by IPA to infer likely upstream regulators (Table 3). Here, analysis is based on comparing an experimental dataset to prior knowledge of transcription factors, microRNAs, kinases and other regulators and their target genes. Regulator “activation” or “inhibition” status was reflected in the sign of the z-score and a z-score threshold of 2 was used for significance. The shared DEGs in both types of senescence identified a common set of predicted upstream regulators including the p21 protein CDKN1A, validating this analysis. Shared regulators included estrogen receptor alpha nuclear hormone receptor ESR1, forkhead box M1 G2/M cell cycle regulatory transcription factor FOXM1, nuclear protein 1 stress response transcriptional regulator NUPR1, and Jarid1B histone H3K4 lysine demethylase KDM5B, each of which has previously been linked to senescence [34–38] but also to diverse other cell responses. In turn, other candidate upstream regulators were identified that were specific to the radiation- or p21-induced senescent samples. Surprisingly, most of the radiation-specific candidate regulators were not DNA damage response factors but linked to other cell signaling pathways.

**Table 3:** Predicted upstream regulators from IPA analysis

Upstream regulator	Gene description
**In IR and p21**	
ESR1	Estrogen receptor
FOXM1	Forkhead Box M1
NUPR1	Nuclear protein, transcriptional regulator 1
KDM5B	Chromobox Homolog 5
CDKN1A	Cyclin Dependent Kinase Inhibitor 1A
**In IR only**	
CBX5	Chromobox Homolog 5
AURKB	Aurora Kinase B
KIAA1524	KIAA1524
ANXA2	Annexin A2
MAPK1	Mitogen-Activated Protein Kinase 1
H2AFY	H2A Histone Family Member Y
EPAS1	Endothelial PAS Domain Protein 1
CCL5	C-C Motif Chemokine Ligand 5
ERBB2	Erb-B2 Receptor Tyrosine Kinase 2
TGFBR2	Transforming Growth Factor Beta Receptor 2
AR	Androgen Recptor
**In p21 only**	
COL18A1	Collagen Type XVIII Alpha 1 Chain
EZH2	Enhancer Of Zeste 2 Polycomb Repressive Complex 2 Subunit
KLF4	Kruppel Like Factor 4

### cmap analysis to identify senescence inducers

**Fig. 5:**
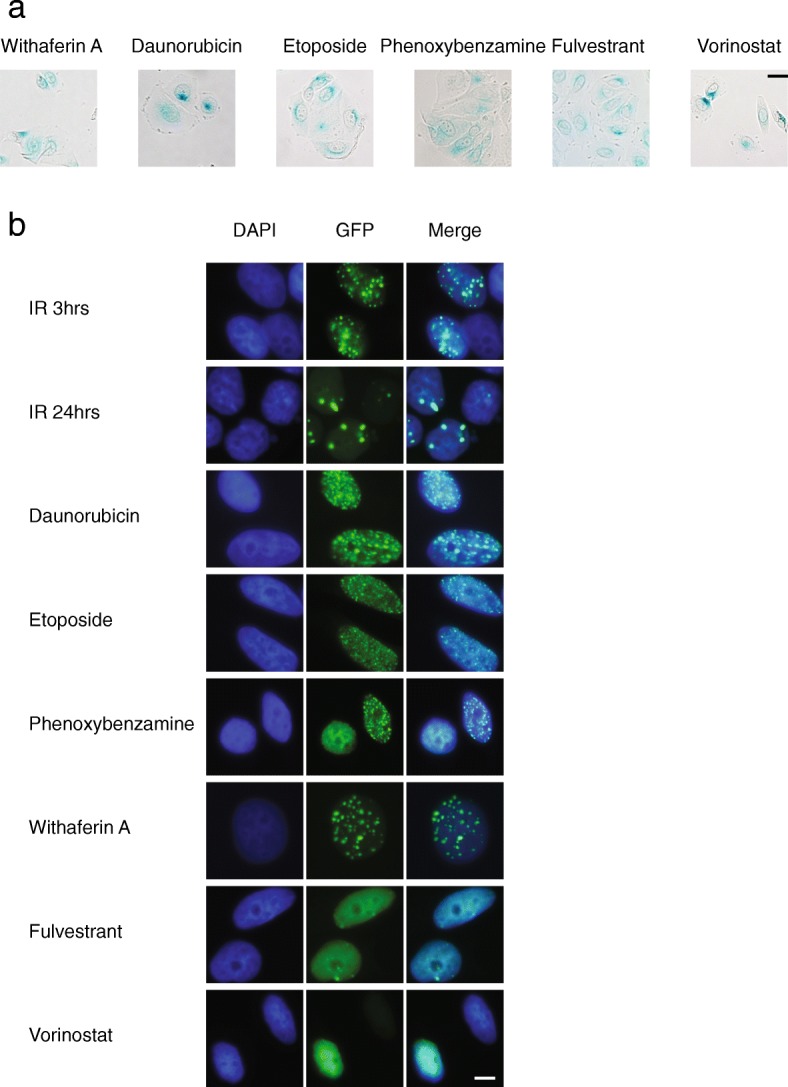
Senescence phenotypes induced by compounds identified by cmap analysis. **a** SA-β-gal assay in MCF7^p21^ cells treated with the cmap screen hits, assessed 5 days after adding the indicated compounds. Scale bar = 20 μm. **b** GFP-IBD foci in MCF7^GFP-IBD^ treated with radiation or cmap screen hits. Nuclear GFP foci indicating double strand breaks were observed at 3 h and 24 h after 6 Gy irradiation. Treatment with daunorubicin, etoposide, withaferin A, or phenoxybenzamine produced nuclear GFP foci at 24 h. Treatment with fulvestrant or vorinostat failed to induce foci above background. Scale bar = 20 μm.

Overall, the results with pathway and upstream regulator analysis failed to confirm that the DEGs shared between radiation- and p21-induced senescence represented a specific signature. As an alternative strategy, we sought to determine if the gene expression pattern represented by the shared DEGs might be able to identify other conditions that induce accelerated senescence. Thus, we queried the Connectivity Map (cmap) database with the 769 shared DEGs toward discovering compounds that induce a similar or reciprocal gene expression pattern. cmap applies a pattern-matching algorithm to compare an input query to a database of microarray analyses of the gene expression effects of treating human cell lines with a diverse set of small molecules. Performing a cmap search based on positive enrichment and limiting analysis to data obtained with MCF7 cells yielded 20 compounds (Table 4). To validate these results, we compared treating MCF7^p21^ cells with the 16 commercially available compounds over a range of concentrations to inducing p21 with doxycycline. Following 5 days of incubation, six of the compounds induced characteristic features of senescence including increased cell size, flattened cell shape, and a positive SA-β-Gal assay (Fig. 5a). The topoisomeraseII poison etoposide (10 μM), the anthracycline daunorubicin (0.1 μM), the steroidal lactone withaferin A (1 μM), the alpha adrenergic receptor blocker phenoxybenzamine (50 μM), the histone deacetylase (HDAC) inhibitor vorinostat (suberoylanilide hydroxamic acid, SAHA, 1 μM), and the selective estrogen receptor degrader fulvestrant (1 μM) each scored as a senescence inducer.

**Table 4:** Top 20 hits identified through the Connectivity Map

Rank	Name	Mean	n	Enrichment	*P*-value	Specificity
1	Etoposide	0.859	2	0.999	0	0
2	Pyrvinium	0.832	4	0.994	0	0
3	Vorinostat	0.595	7	0.952	0	0.1101
6	15-delta prostaglandin	0.635	8	0.828	0	0.0299
7	Trichostatin A	0.519	92	0.776	0	0.1137
8	Thioridazine	0.507	11	0.703	0	0.0233
9	Sirolimus	0.288	25	0.631	0	0.0241
11	Puromycin	0.724	2	0.996	0.00002	0.0215
12	Phenoxybenzamine	0.709	3	0.992	0.00002	0.0394
13	Trifluoperazine	0.514	9	0.724	0.00002	0.0337
15	Rottlerin	0.632	3	0.972	0.00004	0.0052
16	Fulvestrant	0.332	21	0.529	0.00004	0.0971
17	Ionomycin	0.663	3	0.96	0.0001	0.0053
18	Withaferin A	0.711	2	0.986	0.00034	0.0212
19	Daunorubicin	0.605	3	0.938	0.0004	0.034
20	Clotrimazole	0.606	3	0.937	0.0004	0.0161
22	Hycanthone	0.658	2	0.985	0.00044	0.0209
24	Niclosamide	0.68	2	0.984	0.00048	0.0101
26	Nortriptyline	0.673	2	0.983	0.00056	0
28	Ivermectin	0.659	2	0.98	0.0007	0.0152

Hits are limited to perturbagens examined with MCF7 and that yielded a positive score. Under the name of the perturbagen, all instances (different concentration or batch of the same perturbagen for treatment) are included and the mean is the arithmetic mean of the connectivity scores for those instances. The list is ranked based on ascending order of *p*-value then ascending order of enrichment

Given that two of the small molecule senescence inducers, etoposide and daunorubicin, were well-characterized genotoxic chemotherapy agents, we treated MCF7^GFP-IBD^ cells with each compound and evaluated formation of DNA damage foci. Along with the two chemotherapy agents, withaferin A and phenoxybenzamine also induced nuclear GFP foci at 24 h (Fig. 5b), much like radiation. Fulvestrant and vorinostat displayed fewer or no foci, suggesting a DNA damage-independent mechanism of senescence induction.

## Discussion

A wide range of stimuli induce senescence in culture or in tissues, typically via a slow transition where cells initially arrested by a stress response fail to recover and then do not die. While most studied in untransformed, normal cells, senescence can be triggered in immortalized and cancer cells as well. So far no unique regulators have been identified that specify onset of senescence rather than cell death, reversible arrest or proliferation. While most reviews point to unrepaired DNA damage as a common feature underlying senescence, the damage itself appears to be neither necessary nor sufficient. Indeed DNA damage is also cited as a determinant of apoptosis, mitotic catastrophe, and diverse other cellular responses.

Diverse senescence-inducing conditions have been shown to activate expression of the stoichiometric CDK inhibitors (CKIs) p16^INK4A^ and/or p21^CIP1/WAF1^ [4]. Indeed, p21 is highly induced upon p53 activation, which is considered to be critical for DNA damage induced senescence, and cells with defects in the p53/p21 and/or Rb/p16 pathways are resistant to senescence. In turn, ectopic expression of either p21 or p16 is sufficient to induce the cell cycle arrest and morphological features of senescence [39, 40]. The effects of p21 and p16 may extend beyond their CDK targets, insofar as small-molecule CDK inhibitors (CKIs) generally fail to induce senescence. Importantly, the CKIs do not induce the characteristic senescent cell secretome, the SASP [14], implicating DNA damage as a specific driver of inflammatory gene expression versus other senescent cell phenotypes.

Building on the pioneering studies that identified common senescence markers such as p21 and/or p16 and SA-βGal, several prior system biology-based efforts to discover signatures of senescence have applied transcriptomics or proteomics to examine replicative senescence [41, 42] and accelerated senescence [43] in cancer cells and in aging organs of human or animals [8, 9]. While these studies have revealed the common pattern of activation of inflammatory pathways linked to the SASP [10, 11, 44, 45], the gene expression patterns that underlie other characteristic features of senescence have remained largely obscure. We sought to explore whether comparing the transcriptomes of senescent cells induced by DNA damage or CKI overexpression might identify a core senescence gene expression pattern. Thus, we compared the differential gene expression induced in the otherwise immortal breast carcinoma cell line MCF7 (ER^+^, INK4A^−^, Caspase 3^−^) by treatment with ionizing radiation or Tet-On inducible expression of p21. Microarray analysis performed 5 days after irradiation or inducing p21 with doxycycline yielded 790 genes, which were differentially expressed under each condition. Of these, 769 (97%) displayed the same direction of change, suggesting that these shared differentially expressed genes (DEGs) might represent a senescence signature. As expected from the proliferative arrest in senescence, systems level analysis of the shared DEGs identified changes in multiple pathways linked to cell cycle progression and DNA metabolism. Overall though, analysis of pathways failed to detect a specific pattern that might distinguish senescence from other cell stress responses.

Prior studies have revealed a critical role for reactive oxygen species (ROS) in p21-dependent senescence [46], potentially mediating the effects of p21 in part via inducing a DNA damage signal. Treatment with N-acetyl cysteine to reduce ROS could block p21 overexpression from inducing senescence in multiple cell lines, including cancer cells [47]. Nonetheless, our gene expression analysis revealed no elevation of oxidative response genes such as SOD2, CAT, GPX2, or PRDX after p21 induction. In turn, adding N-acetyl cysteine failed to prevent p21 from inducing senescence in the MCF7 cells (data not shown). Although multiple pathways linked to DNA damage response were enriched by the DEGs shared between p21 overexpression and irradiation, examining the specific DEGs that had populated the DDR pathways showed that most were linked to cell cycle progression or arrest rather than DNA repair per se. In turn, the few shared DEGs with an established role in DNA damage repair such as RAD51 and BARD1 displayed *decreased* expression compared to controls.

As a complementary strategy, we queried IPA to identify potential upstream regulatory factors for the DEGs from the radiation- and p21-induced senescent cells. Among factors common to both conditions were CDKN1A, ESR1, FOXM1, NUPR1, and KDM5B. CDKN1A is p21, validating the approach, and ESR1 is estrogen receptor alpha, likely reflecting our use of ER^+^ MCF7 human mammary carcinoma cells. KDM5B is the Jarid1D H3K4 demethylase, previously linked to senescence via the Rb pathway [34, 48, 49]. FOXM1 is a proliferation-associated transcription factor [36] which antagonizes senescence. NUPR1 is a chromatin-binding protein that confers stress resistance. NUPR1 was found to modulate K-RAS-induced senescence and regulate genome-wide DNA methylation [35]. While KDM5B, FOXM1 and NUPR1 are clearly involved in other processes, these regulators may well play significant roles in control of senescence. However, a prior analysis comparing replicative senescence and accelerated senescence by Kural et al. [50] identified a distinct group of common transcription factors, consistent with the overall uncertainties of this strategy.

As an alternative strategy, we sought a means to determine if recapitulating the pattern of gene expression described by the shared DEGs might be sufficient to induce senescence. Here, we took advantage of the Connectivity Map (cmap) database of transcriptional responses to small molecules [51–53]. By searching a query pattern of gene expression against the cmap library, it is possible to identify existing molecules whose effects phenocopy a physiological response, an approach with broad applications for discovery of chemical probes and drugs. To examine the potential to find senescence inducing agents, we queried the database with the shared DEGs to identify compounds that induce similar gene expression patterns in MCF7 cells. Of the top 20 compounds identified by cmap analysis, we obtained 16 and found that six could indeed induce senescence. Four compounds phenocopied radiation-induced senescence by inducing DNA damage foci after 1 day and then displayed senescent morphology and SA-β-Gal activity after 5 days. Two of these agents, the semisynthetic podophyllotoxin derivative etoposide and the natural product anthracycline daunorubicin, are well-characterized genotoxic agents that have long been considered topoisomerase II poisons [54, 55]. Multiple epipodophyllotoxins and anthracyclines are effective senescence inducers in MCF7, validating the cmap screen. The two other hits that induced DNA damage were unanticipated. The natural product steroidal lactone withaferin A is toxic to tumor cell lines and has been linked to production of reactive oxygen species, providing a potential source of DNA damage. The alpha-adrenergic antagonist phenoxybenzamine has not been linked to DNA damage, but we only observed these effects at 50 μM, well above the concentrations used in most studies. Considering the DNA damage foci observed with all four of these agents, the most parsimonious explanation may be that any agent that promotes DNA damage foci that persist for a day or more has potential as a senescence inducer. Our prior studies [23, 56, 57] suggest that such compounds are relatively rare, except among agents that are already known to induce chromosomal DNA damage on their own, such as chemotherapy agents.

Two other agents induced senescence within 5 days, but without dramatically increasing DNA damage foci. Vorinostat (SAHA) is a histone deacetylase (HDAC) inhibitor that inhibits both class I and class II HDACs. SAHA was shown to induce a senescence-like arrest in MCF7 cells [58] which may reflect increased expression of p21 [59]. The last hit, fulvestrant, is a selective estrogen receptor degrader (SERD). Significantly, knockdown of ER in MCF7 cells blocks proliferation in part via derepression of p21 [37], suggesting that fulvestrant may mediate its effect by a similar mechanism. Despite this result and our finding ESR1 as a candidate upstream regulator of senescence, given the wide range of estrogen receptor-dependent genes, fulvestrant is not particularly promising as a chemical probe for senescence.

## Conclusions

Here, we identified differentially expressed genes (DEGs) shared among senescent MCF7 breast carcinoma cells after irradiation or overexpression of the p21^WAF1/CIP1^ protein and then applied system biology methods to explore the shared DEGs as a candidate senescence signature. The analysis highlighted multiple previously annotated pathways related to cell proliferation, consistent with the cell cycle arrest in senescent cells. A complementary analysis of upstream regulators revealed multiple factors previously implicated in senescence, further validating the strategy. Systems analysis also confirmed that senescence induced by p21 overexpression lacks the inflammatory signaling prominent in senescence induced by radiation. As a final approach to validation, we queried the cmap database of drug response patterns with the shared DEGs and identified multiple compounds that could induce senescence on their own. Taken together, these results suggest that senescence induced by radiation or p21 expression share a core gene expression pattern linked to key features of the senescent phenotype. The discovery of small molecule senescence inducers by querying cmap with this pattern may point to having identified a signature of cell stress upstream of senescence rather than the cell state per se. Further analysis may identify novel targets to modulate therapy-induced senescence in cancer treatment.

## Additional files

Additional file 1: Python script to calculate the average gene expression. (PY 683 bytes)

Additional file 2: The differentially expressed genes shared between IR- and p21-induced senescence. (XLSX 38 kb)

Additional file 3: Differentially expressed genes in interferon signaling pathway in IR-induced senescence. (XLS 28 kb)

## Abbreviations

CKI: CDK inhibitors
cmap: Connectivity Map
DAVID: Database for Annotation, Visualization and Integrated Discovery
DEG: Differentially expressed genes
DSB: Double strand break
GO: Gene Ontology
HDAC: Histone deacetylase
IPA: Ingenuity Pathway Analysis
IR: Ionizing radiation
IRIF: Ionizing radiation induced foci
KEGG: Kyoto Enrichment of Genes and Genomics
ROS: Reactive oxygen species
RS: Replicative senescence
SAM: Significance Analysis of Microarrays
SASP: Senescence-associated secretory phenotype
SA-β-Gal: Senescence-associated β-galactosidase activity
SERD: Selective estrogen receptor degrader
SIPS: Stress-induced premature senescence
TIS: Therapy-induced senescence
